# Discontinuation of adjuvant hormone therapy among breast cancer patients not previously attending mammography screening

**DOI:** 10.1186/s12916-019-1252-6

**Published:** 2019-01-31

**Authors:** Wei He, Louise Eriksson, Sven Törnberg, Fredrik Strand, Per Hall, Kamila Czene

**Affiliations:** 1 Department of Medical Epidemiology and Biostatistics, Karolinska Institutet, Nobels väg 12 A, 171 77 Stockholm, Sweden; 2 Department of Oncology-Pathology, Cancer Center Karolinska, Department of Oncology, Radiumhemmet, Karolinska Institutet and Karolinska University Hospital, Stockholm, Sweden; 3 Department of Cancer Screening, Stockholm-Gotland Regional Cancer Centre, Stockholm, Sweden; 4 Department of Thoracic Radiology, Karolinska University Hospital, Stockholm, Sweden; 5 Department of Oncology, South General Hospital, Stockholm, Sweden

**Keywords:** Breast cancer, Mammography screening, Discontinuation of adjuvant hormone therapy, Disease-free survival

## Abstract

**Background:**

Breast cancer patients who have not previously attended mammography screening may be more likely to discontinue adjuvant hormone therapy and therefore have a worse disease prognosis.

**Methods:**

We conducted a population-based cohort study using data from Stockholm Mammography Screening Program, Stockholm-Gotland Breast Cancer Register, Swedish Prescribed Drug Register, and Swedish Cause of Death Register. Women in Stockholm who were diagnosed with breast cancer between 2001 and 2008 were followed until December 31, 2015. Non-participants of mammography screening were defined as women who, prior to their breast cancer diagnosis, were invited for mammography screening but did not attend.

**Results:**

Of the 5098 eligible breast cancer patients, 4156 were defined as screening participants and 942 as non-participants. Compared with mammography screening participants, non-participants were more likely to discontinue adjuvant hormone therapy, with an adjusted hazard ratio (HR) of 1.30 (95% CIs, 1.11 to 1.53). Breast cancer patients not participating in mammography screening were also more likely to have worse disease-free survival, even after adjusting for tumor characteristics and other covariates (adjusted HR 1.22 (95% CIs, 1.05 to 1.42 for a breast cancer event).

**Conclusions:**

Targeted interventions to prevent discontinuation of adjuvant hormone therapy are needed to improve breast cancer outcomes among women not attending mammography screening.

**Electronic supplementary material:**

The online version of this article (10.1186/s12916-019-1252-6) contains supplementary material, which is available to authorized users.

## Background

Breast cancer is the most frequently diagnosed cancer and the second leading cause of cancer deaths among women, accounting for 15% of total cancer deaths worldwide and 13% of total cancer deaths in Sweden in 2012 [1, 2]. Mammography screening, which is shown to lower the risk of breast cancer mortality by over 20% [3–5], is one of the most important steps that women can take to reduce breast cancer mortality. Despite such demonstrated benefit, 25–42% of women do not participate in mammography screening programs [6–9].

Adjuvant hormone therapy—using tamoxifen and aromatase inhibitors—has been reported to lower the risk of breast cancer recurrence by over 30% [10]. Despite this, over half of breast cancer patients discontinue such treatment [11–13]. Discontinuation of adjuvant hormone therapy reduces treatment efficacy [14–18], resulting in increased cancer recurrence and mortality, which could otherwise be prevented.

Previous studies have shown that barriers to mammography screening adherence may also prevent patients from adhering to subsequent adjuvant hormone therapy [12, 19–21]. We tested the hypothesis that, compared to mammography screening participants, screening non-participants are more likely to discontinue adjuvant hormone therapy, and have a worse breast cancer prognosis, even after adjusting for tumor characteristics.

## Methods

### Data sources

This study was approved by the Regional Ethical Review Board in Stockholm, Sweden (Approval number: 2009/254-31/4, 2011/2010-32).

Since 1989, The Stockholm Mammography Screening Program invited all women in Stockholm aged 50 to 69 years for mammography screening at 24-month intervals, and since 2005, women aged 40 to 49 years have been invited at 18-month intervals [22–24]. The Stockholm-Gotland Breast Cancer Register includes all breast cancers diagnosed in Stockholm since 1976. This register, with a completeness of 98% [25, 26], includes data on diagnosis, tumor characteristics, surgery, postoperative treatment, and follow-up. The Swedish Prescribed Drug Register contains detailed information for all drugs prescribed and dispensed to the Swedish population since July 1, 2005 [27].

### Study population

**Fig. 1:**
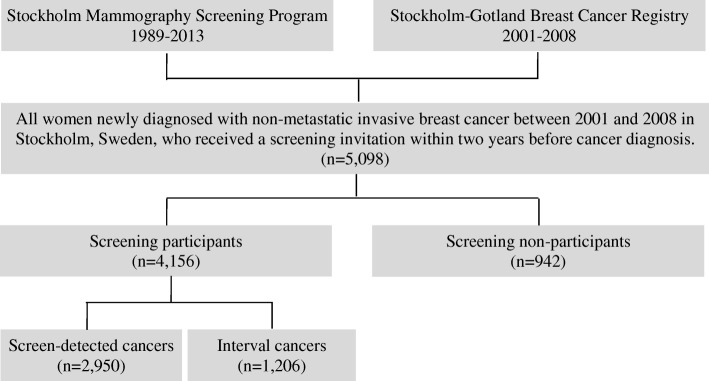
Flow chart of study participants

Using the unique personal identification number [28], we linked the Stockholm-Gotland Breast Cancer Register to the Stockholm Mammography Screening Program data. Through this linkage, we identified 5855 women in Stockholm who were diagnosed with breast cancer between 2001 and 2008 and who were invited to a mammography screening 2 years (or 18 months for those aged 40–49 years) before their breast cancer diagnosis. We excluded patients with in situ cancer (*n* = 680) and with distant metastasis at cancer diagnosis (*n* = 77), leaving a total of 5098 patients for the final analysis (Fig. 1).

Given the Swedish Prescribed Drug Register was established in July 2005, our analysis of discontinuation of adjuvant hormone therapy was restricted to 1966 patients who were diagnosed with breast cancer after this date and who had initiated adjuvant hormone therapy with at least one prescription of tamoxifen (ATC codes L02BA01) or aromatase inhibitors (ATC codes L02BG).

### Exposure definition

Non-participants of mammography screening were defined as women who were invited for mammography screening 2 years (or 18 months, for those aged 40–49 years) before their breast cancer diagnosis but did not attend. The above cutoffs were used given that this is the longest screening interval applied in the Stockholm Mammography Screening Program throughout the study period [22–24].

### Covariates

Information on age at diagnosis, menopausal status at diagnosis, family history of breast cancer, tumor size, lymph node involvement, estrogen receptor status, progesterone receptor status, HER2 status, and tumor grade was retrieved from the Stockholm-Gotland Breast Cancer Register. Information on country of birth and marital status at diagnosis was retrieved from the Total Population Register [29]. Information on comorbidities used to calculate the Charlson Comorbidity Index was retrieved from the Swedish Patient Register [30]. Information on education, employment status, cigarette smoking, and parity was retrieved from a questionnaire survey completed by a subgroup of patients (60%). Detailed information on this survey can be found elsewhere [12, 23, 31].

### Outcome definitions

#### Discontinuation of adjuvant hormone therapy

Discontinuation of adjuvant hormone therapy was defined as having any interval between two consecutive dispenses of greater than 180 days during the follow-up [12, 31]. In Sweden, a 3-month supply is the maximum allowed to be dispensed at each time. Given this, an interval of more than 180 days indicates that at least two dispenses have been missed, resulting in a shortage of the drug. Patients who changed between tamoxifen and aromatase inhibitors were defined as continuers, as long as they remained on adjuvant hormone therapy.

Discontinuation was defined by monitoring patients from the first prescription of tamoxifen or aromatase inhibitors until death, local recurrence, distant metastasis, contralateral breast cancer, emigration, end of study period (December 31, 2015), or completion of a 5-year treatment, whichever came first. Time to discontinuation was calculated as the interval between the first and last prescription, added to the number of days of supply from the last prescription.

#### Breast cancer events

Patients were monitored from the date of cancer diagnosis until death, emigration, or end of study period (December 31, 2015), whichever came first. Information on local recurrence, distant metastasis, and contralateral breast cancer was retrieved from the Stockholm-Gotland Breast Cancer Registry. Information on date of emigration was retrieved from the Swedish Emigration Registry. Information on cause of death was retrieved from the Swedish Cause of Death Registry.

Time to breast cancer event was defined as the time from cancer diagnosis to local recurrence, distant metastasis, contralateral breast cancer (> 3 months after the primary breast cancer), or death from breast cancer, whichever came first [31].

### Statistical analyses

Chi-square tests (or Fisher’s exact test if the expected cell frequencies were less than 10) were used to compare differences in baseline and tumor characteristics among screening participants versus non-participants. Only characteristics with a *p* value < 0.05 were included in the multivariable analyses. Kaplan-Meier analysis and Cox regression analysis were used to compare differences in discontinuation of adjuvant hormone therapy and breast cancer events among screening participants versus non-participants. The proportionality assumption for running a Cox model was checked using the Schoenfeld residual test, with no model violation observed.

We repeated our analyses by comparing screening non-participants with subgroups of screening participants: patients with screen-detected cancers (cancer detected after a positive screening result) and interval cancers (cancer detected after a negative screening mammography but before the next scheduled examination), given that these are both diverse groups with different tumor characteristics and breast cancer outcomes.

We also repeated our analysis of screening non-participation and breast cancer events using a competing risk regression model, accounting for non-breast cancer deaths as a competing event.

All statistical analyses were two-sided and performed using SAS version 9.4 (SAS Institute, Cary, NC) or Stata version 13.0 (Stata Corporation, College Station, TX). Statistical significance was determined at *p <* 0.05.

## Results

### Baseline characteristics

Table 1 summarizes the characteristics of the 4156 screening participants and 942 non-participants. Compared with mammography screening participants, non-participants were more likely to be non-Nordic and divorced.

**Table 1:** Characteristics of women diagnosed with breast cancer in Stockholm, Sweden, 2001–2008, screening non-participants vs participants

	Mammography screening		*p* value
	Participants	Non-participants	
Register data (*n* = 5098)			
Age, years^a^			< .001
40–49	161 (3.9)	23 (2.4)	
50–59	1879 (45.2)	526 (55.8)	
≥ 60	2116 (50.9)	393 (41.7)	
Menopausal status			0.115
Pre-menopause	554 (14.3)	108 (12.3)	
Post-menopause	3313 (85.7)	771 (87.7)	
Unknown	289	63	
Country of birth			0.007
Nordic	4068 (97.9)	908 (96.4)	
Non-Nordic	88 (2.1)	34 (3.6)	
Marital status			< .001
Married	2220 (54.3)	449 (49.4)	
Unmarried	593 (14.5)	133 (14.6)	
Widowed	299 (7.3)	52 (5.7)	
Divorced	976 (23.9)	274 (30.2)	
Unknown	68	34	
Charlson Comorbidity Index			0.235
0	3649 (87.8)	829 (88.0)	
1	374 (9.0)	92 (9.8)	
≥ 2	133 (3.2)	21 (2.2)	
Family history of breast cancer			0.417
No	782 (28.4)	193 (30.0)	
Yes	1975 (71.6)	451 (70.0)	
Unknown	1399	298	
Questionnaire data (*n* = 3038)			
Education, years			0.639
≤ 9	374 (19.1)	70 (18.2)	
9–12	553 (28.2)	102 (26.5)	
> 12	1033 (52.7)	213 (55.3)	
Other	488	80	
Unknown	104	21	
Employment status			0.175
Employed	1666 (68.3)	332 (72.5)	
Unemployed (≥ 6 months)	35 (1.4)	6 (1.3)	
Retired	626 (25.7)	96 (21.0)	
Long-term sick leave (≥ 6 months)	65 (2.7)	17 (3.7)	
Housewife	47 (1.9)	7 (1.5)	
Others or unknown	113	28	
Cigarette smoking			0.634
Never	942 (38.3)	173 (37.1)	
Ever	1518 (61.7)	293 (62.9)	
Unknown	92	20	
Parity			0.971
0	385 (15.6)	71 (15.2)	
1–2	1549 (62.6)	295 (63.2)	
≥ 3	540 (21.8)	101 (21.6)	
Unknown	78	19	

^a^The Stockholm Mammography Screening Program invites women aged 40–49 years only from mid-2005

### Tumor characteristics

**Fig. 2:**
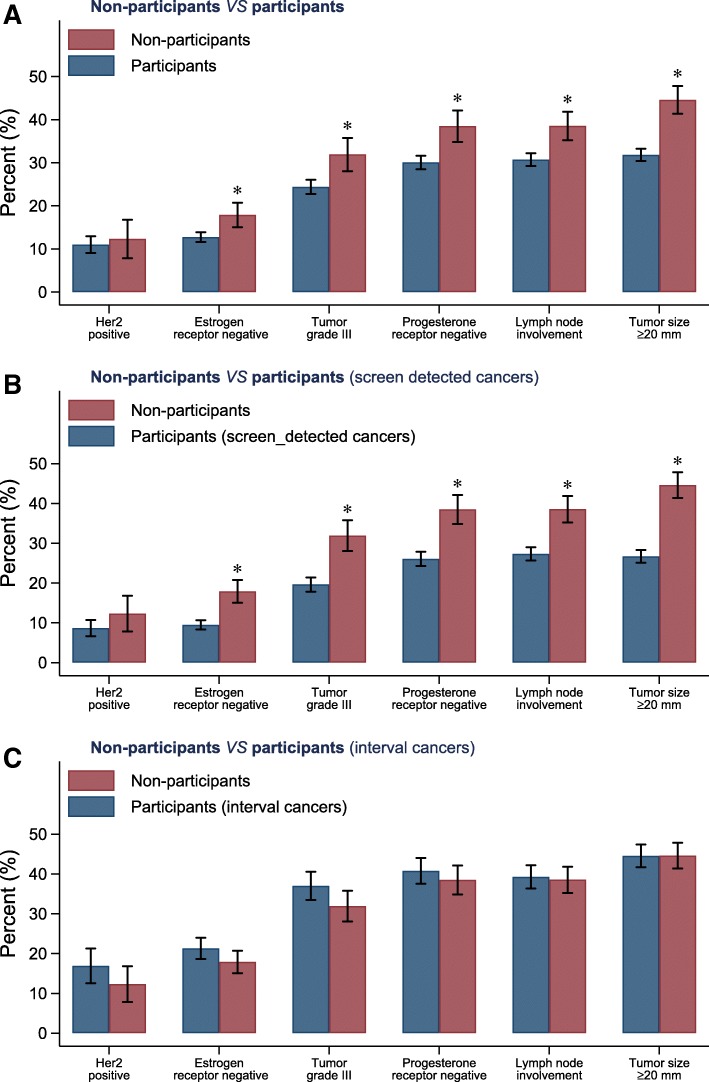
Tumor characteristics of women diagnosed with breast cancer in Stockholm, Sweden, 2001–2008. **a** Screening non-participants vs participants. **b** Screening non-participants vs participants diagnosed with screen-detected cancers. **c** Screening non-participants vs participants diagnosed with interval cancers. *p < 0.05 for comparison between the groups

Figure 2 shows that non-participants were more likely to be diagnosed with larger tumors (≥ 20 mm diameter), to have positive lymph nodes, to have estrogen- and progesterone receptor-negative tumors, and to have tumors of a higher grade. From a prognostic point of view, worse tumor characteristics were only found when comparing non-participants to screening participants who were diagnosed with screen-detected cancers (Fig. 2). In contrast, non-participants had similar, or even more favorable, tumor characteristics when compared to screening participants who were diagnosed with interval cancers (Fig. 2).

### Discontinuation of adjuvant hormone therapy

**Fig. 3:**
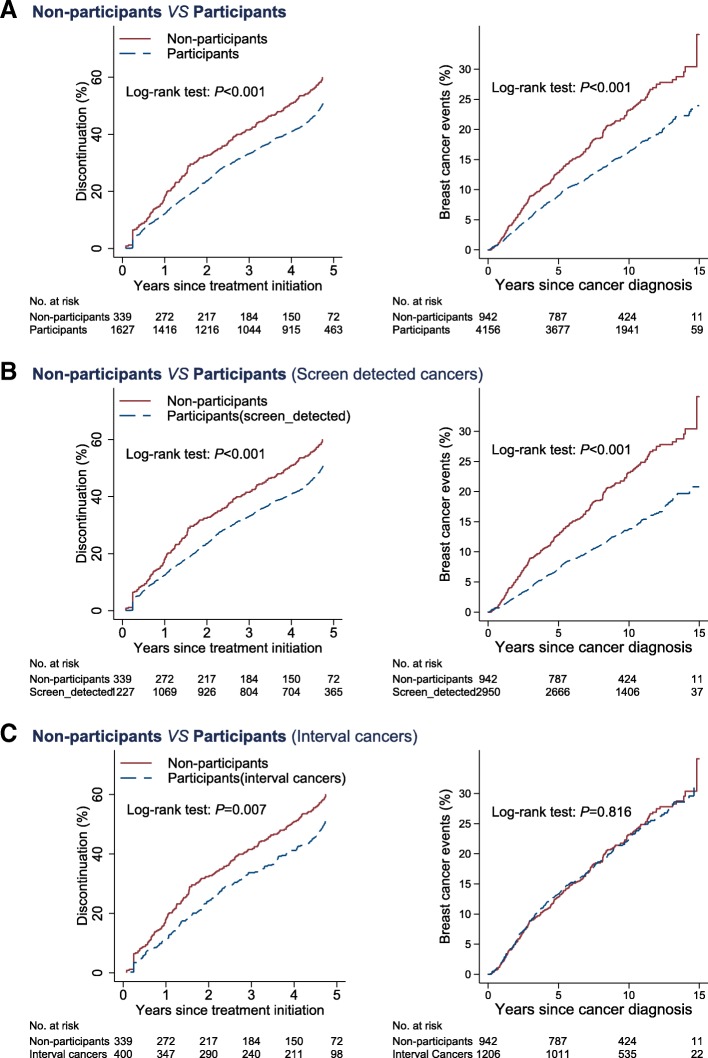
Discontinuation of adjuvant hormone therapy (left column) and breast cancer events (right column) in women diagnosed with breast cancer in Stockholm, Sweden, 2001–2008. **a** Screening non-participants vs participants. **b** Screening non-participants vs participants diagnosed with screen-detected cancers. **c** Screening non-participants vs participants diagnosed with interval cancers

Figure 3 shows that, compared to mammography screening participants, non-participants were more likely to discontinue adjuvant hormone therapy. The 5-year discontinuation rate was 50.9% (95% CI, 48.4 to 53.4%) among screening participants and 60.0% (95% CI, 54.6 to 65.4%) among non-participants. Further adjustment for other covariates did not change these estimates, with an adjusted hazard ratio (HR) of 1.30 (95% CI 1.11 to 1.53) for non-participants versus screening participants (Table 2).

**Table 2:** Discontinuation of adjuvant hormone therapy and breast cancer events in women diagnosed with breast cancer in Stockholm, Sweden, 2001–2008. (A) Screening non-participants vs participants, (B) screening non-participants vs participants diagnosed with screen-detected cancers, and (C) screening non-participants vs participants diagnosed with interval cancers

	Discontinuation of adjuvant hormone therapy, no. (%)		Multivariable-adjusted, HR^a^ (95% CIs)	Breast cancer events, no. (%)		Multivariable-adjusted, HR^a^ (95% CIs)
	No	Yes		No	Yes	
(A) Non-participants vs participants						
Participants	822 (50.5)	805 (49.5)	1.00 (reference)	3449 (83.0)	707 (17.0)	1.00 (reference)
Non-participants	146 (43.1)	193 (56.9)	1.30 (1.11–1.53)	719 (76.3)	223 (23.7)	1.22 (1.05–1.42)
(B) Non-participants vs participants (screen-detected cancers)						
Participants (screen-detected cancers)	615 (50.1)	612 (49.9)	1.00 (reference)	2523 (85.5)	427 (14.5)	1.00 (reference)
Non-participants	146 (43.1)	193 (56.9)	1.31 (1.10–1.54)	719 (76.3)	223 (23.7)	1.32 (1.12–1.57)
(C) Non-participants vs participants (interval cancers)						
Participants (interval cancers)	207 (51.8)	193 (48.2)	1.00 (reference)	926 (76.8)	280 (23.2)	1.00 (reference)
Non-participants	146 (43.1)	193 (56.9)	1.25 (1.02–1.53)	719 (76.3)	223 (23.7)	1.05 (0.88–1.25)

^a^Hazard ratio (HR) and 95% confidence intervals (95% CIs) adjusted for age, country of birth, marital status, tumor size, lymph node involvement, estrogen receptor status, progesterone receptor status, and grade

Consistently higher discontinuation rates were found when comparing non-participants with subgroups of screening participants: an adjusted HR of 1.31 (95% CI, 1.10 to 1.54) when compared to screen-detected cancers and 1.25 (95% CI, 1.02 to 1.53) when compared to interval cancers (Table 2).

### Breast cancer events

Figure 3 shows that, compared to mammography screening participants, non-participants were more likely to have a worse disease-free survival. The 10-year cumulative risk of being diagnosed with a breast cancer event (local recurrence, distant metastasis, contralateral breast cancers, or death from breast cancer) was 16.2% (95% CI, 15.1 to 17.4%) among screening participants and 23.2% (95% CI, 20.5 to 26.2%) among non-participants. Further adjusting for tumor characteristics and other covariates explained part, but not all, of the association between non-participation and breast cancer events (adjusted HR 1.22 (95% CI 1.05 to 1.42)) (Table 2).

Consistently higher rates of breast cancer events were found when comparing non-participants with screening participants diagnosed with screen-detected cancers (Table 2). However, when compared to screening participants diagnosed with interval cancers, non-participants had a similar risk of breast cancer events (adjusted HR 1.05 (95% CI 0.88 to 1.25)) (Fig. 3, Table 2).

Sensitivity analyses using competing risk regression models provided similar results (Additional file 1: Table S1).

## Discussion

To our best knowledge, this is the first study showing that prior non-adherence to mammography screening is associated with subsequent non-adherence to adjuvant hormone therapy among breast cancer patients. Specifically, we found that, compared to mammography screening participants, non-participants were more likely to discontinue adjuvant hormone therapy and to have a worse breast cancer prognosis, even after adjusting for tumor characteristics.

We found that screening non-participants represent a subgroup of breast cancer patients who are more likely to discontinue adjuvant hormone therapy. This is in line with previous studies which show that age, marital status, and other patient-related characteristics are associated with both screening non-adherence and discontinuation of adjuvant hormone therapy [11, 12, 19, 21]. This may mean that women who do not attend mammography screening are less likely to remain on adjuvant hormone therapy due to shared barriers and mechanisms. Given validation, these findings are of clinical importance since non-adhering women would most likely benefit from targeted interventions.

Women not participating in mammography screening have been shown to have a worse survival than screening participants [4, 32], largely attributable to having worse tumor characteristics [4, 33, 34]. Our study confirmed and extended these findings by showing that poorer outcomes persisted even after adjustment for tumor characteristics and other known confounders. This residual survival disadvantage is modest but statistically significant and is likely to be partly due to discontinuation of adjuvant hormone therapy.

Interval cancers have been reported to have worse tumor characteristics than screen-detected cancers [23, 32–34]. However, previous studies have usually compared interval cancers with screen-detected cancers [23, 35]. Our study provides further evidence by comparing interval cancers with cancers detected among screening non-participant, showing that interval cancers may have worse tumor characteristics. However, despite that, interval cancers have been reported to have a similar survival as cancers diagnosed among women not participating in screening [36, 37]. This observation is somewhat contradictory to the common belief that interval cancers have a more aggressive molecular phenotype and a faster growth rate [23, 32, 38]. This also challenges the theory of a strong correlation between growth rate and metastatic potential, and the belief that patients with interval cancers should receive more aggressive treatment [36, 37]. We confirmed and extended the findings of previous studies, showing that interval cancers have similar breast cancer outcomes when compared with screening non-participants. However, we found that screening participants diagnosed with interval cancers had better treatment adherence than women not participating in screening. It is therefore possible that interval cancers have a more aggressive tumor biology than cancers in women not participating in screening, but this difference, which is not observed when investigating survival, is masked by better treatment adherence among screening attendees. Thus, interpretation of survival data comparing interval cancers to cancers diagnosed in screening non-participants may be misleading, unless treatment adherence is taken into consideration.

Our study has certain limitations. Firstly, misclassification of exposure is possible, given that women defined as screening non-participants may have undergone opportunistic screening at private hospitals [39]. Secondly, it may be possible—but very rare in Sweden based on radiologists’ experience—that women defined as screening participants may have coincidentally had a self-detected lump before the invited screening (thus constituting a diagnostic rather than screening mammogram). We believe that such misclassification would likely dilute the observed associations. Thirdly, we were unable to investigate the association between screening non-participation and other forms of treatment, due to a lack of data on adherence to radiotherapy and chemotherapy. However, almost all women in Sweden with breast cancer will adhere to radiotherapy and chemotherapy, except for those with severe treatment-related side effects (personal communication with clinicians). Finally, we lacked information on some measures of socioeconomic status, such as household income. However, we do not believe this is a large issue, given that Swedish healthcare, including both mammography screening and breast cancer treatments, is publically financed. Additionally, previous studies have found that being single or non-employed was the most important socioeconomic predictor of screening non-attendance in Sweden, which were adjusted for in this study.

## Conclusions

In conclusion, we found that screening non-participants represent a subgroup of breast cancer patients who are more likely to discontinue adjuvant hormone therapy. We have thus defined screening non-participants as a high-risk, but currently neglected, population for treatment non-adherence. These women would benefit from targeted interventions to prevent discontinuation of adjuvant hormone therapy.

## Additional file

Additional file 1: **Table S1.** Hazard ratio (HR) and 95% confidence intervals (95% CIs) for discontinuation of adjuvant hormone therapy and breast cancer events derived from competing risk regression models. Discontinuation of adjuvant hormone therapy and breast cancer events in women diagnosed with breast cancer in Stockholm, Sweden, 2001–2008. (A) Screening non-participants vs participants; (B) screening non-participants vs participants diagnosed with screen-detected cancers; (C) screening non-participants vs participants diagnosed with interval cancers. Hazard ratio (HR) and 95% confidence intervals (95% CIs) were derived from competing risk regression models. (DOCX 13 kb)

## Abbreviations

ATC: Anatomical Therapeutic Chemical Classification System
CI: Confidence interval
HR: Hazard ratio
