# Supplementary material for: Discontinuation of adjuvant hormone therapy among breast cancer patients not previously attending mammography screening
Source: BMC Med. 2019 Jan 31;17:24. doi: 10.1186/s12916-019-1252-6 (PMC6354407; doi:10.1186/s12916-019-1252-6)
Supplement: Supplementary file 1 — Table S1. Hazard ratio (HR) and 95% confidence intervals (95% CIs) for discontinuation of adjuvant hormone therapy and breast cancer events derived from competing risk regression models. Discontinuation of adjuvant hormone therapy and breast cancer events in women diagnosed with breast cancer in Stockholm, Sweden, 2001–2008. (A) Screening non-participants vs participants; (B) screening non-participants vs participants diagnosed with screen-detected cancers; (C) screening non-participants vs participants diagnosed with interval cancers. Hazard ratio (HR) and 95% confidence intervals (95% CIs) were derived from competing risk regression models. (DOCX 13 kb) [file 12916_2019_1252_MOESM1_ESM.docx]

**Table S1**. Discontinuation of adjuvant hormone therapy and breast cancer events in women diagnosed with breast cancer in Stockholm, Sweden, 2001-2008. (A) Screening non-participants *VS* participants; (B) screening non-participants *VS* participants diagnosed with screen-detected cancers; (C) screening non-participants *VS* participants diagnosed with interval cancers.

|  | Breast cancer events  No.(%) | | Multivariable-  Adjusted^†^  HR^*^(95%CIs) |
| --- | --- | --- | --- |
|  | No | Yes |  |
| **(A)Non-participants VS participants** |  |  |  |
| Participants | 3449(83.0) | 707(17.0) | 1.00(Reference) |
| Non-participants | 719(76.3) | 223(23.7) | 1.20(1.03-1.40) |
| **(B)Non-participants VS participants** (screen-detected cancers) |  |  |  |
| Participants (screen-detected cancers) | 2523(85.5) | 427(14.5) | 1.00(Reference) |
| Non-participants | 719(76.3) | 223(23.7) | 1.29(1.09-1.53) |
| **(C)Non-participants VS participants**(Interval cancers) |  |  |  |
| Participants (Interval cancers) | 926(76.8) | 280(23.2) | 1.00(Reference) |
| Non-participants | 719(76.3) | 223(23.7) | 1.04(0.87-1.24) |

^*^ Hazard ratio (HR) and 95% confidence intervals (95% CIs) derived from competing risk regression models, adjusted for age, country of birth, marital status, tumor size, lymph node involvement, estrogen receptor status, progesterone receptor status, and tumour grade.

^†^ After accounting for non-breast cancer death as a competing event.
